# A pilot study to assess compliance and impact of health warnings on tobacco products in the Udupi district of Karnataka State, India

**DOI:** 10.18332/tid/105894

**Published:** 2019-05-25

**Authors:** Somya Mullapudi, John Britton, Muralidhar M. Kulkarni, Crawford Moodie, Veena G. Kamath, Asha Kamath

**Affiliations:** 1 Department of Community Medicine, Kasturba Medical College, Manipal Academy of Higher Education, Manipal, India; 2 UK Centre for Tobacco and Alcohol Studies, Division of Epidemiology and Public Health, University of Nottingham, Nottingham, United Kingdom; 3 Institute for Social Marketing, University of Stirling, Stirling, United Kingdom; 4 Department of Statistics, Prasanna School of Public Health, Manipal Academy of Higher Education, Manipal, India

**Keywords:** compliance, impact, health warnings, COTPA, tobacco

## Abstract

**INTRODUCTION:**

The Government of India has taken several steps to reduce tobacco use, including legislation in the Cigarettes and Other Tobacco Products Act (COTPA) requiring health warnings on tobacco products. This study assessed compliance with the legislation on warnings, and awareness of these warnings and their perceived impact in preventing tobacco uptake among college students in a district of Karnataka, India.

**METHODS:**

This study consisted of two components, pack collection and a survey. For the first, tobacco packs were obtained from all tobacco selling shops in an urban and a rural locality in the Karkala block of Udupi district. Empty cigarette packs were collected from shops, and full packs were purchased if empty packs were not available . The packs were collected to measure their dimensions, as per the Tobacco Pack Surveillance System guidelines, and assessed for compliance, as per COTPA. For the second component of the study, a questionnaire was distributed to each college student to fill in; this was done to assess awareness of the new warnings at the time of the pilot survey, knowledge of harms, and perceptions of the warnings in reducing tobacco uptake.

**RESULTS:**

We collected 26 tobacco packs. Two (8%) packs had warnings that were the correct size (85% of the main display areas), 15 (58%) packs had clear and legible warnings, and 18 (69%) packs had warning messages in the appropriate language. In the student survey, 60% of males and 52% of females indicated that they would not start using any tobacco products on seeing the new warnings.

**CONCLUSIONS:**

Only a few studies other than our pilot study have assessed compliance with legislation on health warnings in low- or middle-income countries. Although health warnings were perceived as a deterrent to tobacco use among students, compliance with national legislation in this pilot study was found to be low.

## INTRODUCTION

Global tobacco use causes an estimated 7 million deaths each year, making tobacco control an international priority^1^. In India, a middle-income country, an estimated 28.6% of adults aged 15 years and older, approximately 267 million people^2^, use some form of tobacco, at an estimated cost to the economy in 2011 of over INR 1044816 million (approximately USD 22.4 billion) or 1.16% of India’s Gross Domestic Product^3^. In Karnataka State in Southern India, the prevalence of tobacco use among adults is 22.8%, equivalent to 12 million users^4^. The Government of India has taken several steps to reduce tobacco use, including legislation contained in the Cigarettes and Other Tobacco Products Act (COTPA). One of these measures is the requirement for tobacco packs to carry both pictorial and text health warnings^5^, which in 2016 was enhanced by new legislation increasing the size of the health warnings on packs from 40% to 85% of the main display areas^6^.

To date, there has been relatively little published research on the effect of, or compliance with, health warnings on tobacco packs in India^7,8^. This is particularly true of the new larger health warnings, though some original work has been published in abstract form^7,8^.

This study was therefore carried out to obtain preliminary data on the extent to which tobacco products sold in a COTPA-compliant district of Karnataka are compliant with the 2016 legislation and to study awareness and perception of the impact of the health warnings among college students in the age range of individuals most vulnerable to initiating tobacco use^2^.

## METHODS

We evaluated tobacco packaging and health warning compliance with Sections 7–9 of COTPA, which deal with size, clarity, legibility, conspicuousness and language of the health warnings^5^, using an observational study design. We visited a sample of convenience shops in one urban and one rural locality in Karkala block of Udupi District and collected examples of packs of all the available brands of tobacco (cigarettes, beedis, oral tobacco and snuff) being sold in these shops. All shops selling tobacco products in the two localities were visited.

**Figure 1:**
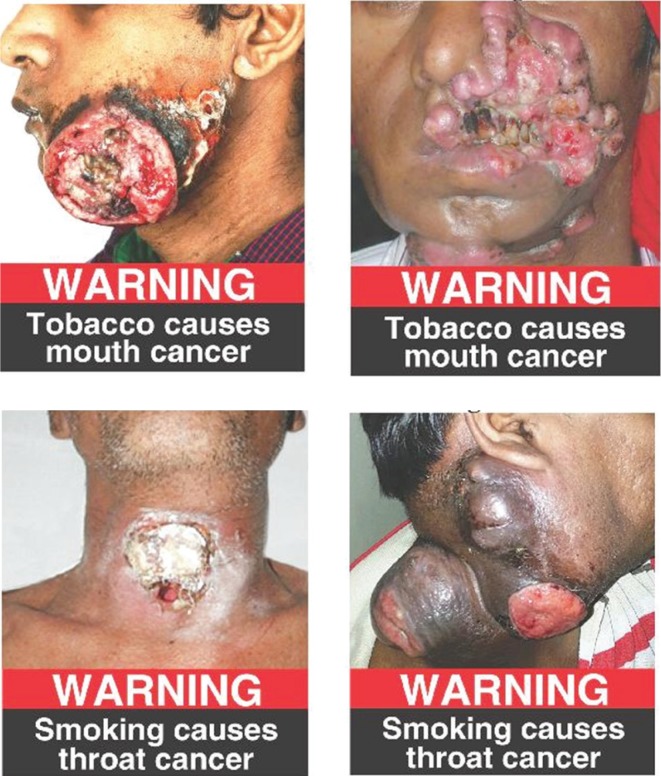
COTPA compliant health warnings

**Figure 2:**
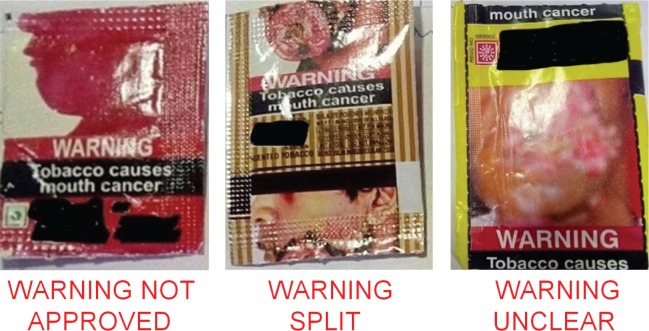
Non-compliant health warnings

The assessment of compliance for the three sections of COTPA was carried out by two researchers separately, with any ambiguity resolved by discussion with a third researcher. For compliance to Section 7, the width/length/circumference/diameter of the pictorial and text warnings were measured in centimetres with the help of a calibrated scale for cuboid packs and measuring tape for conical and cylindrical packs. For packs with bevelled edges, the warnings were measured as per the TPackSS (Tobacco Pack Surveillance System) guidelines developed by the Johns Hopkins Bloomberg School of Public Health^9^. The readings were then compared and similar measurements, to the nearest of 0.1 centimetre, were accepted as the final reading. Health warnings were deemed to be compliant if they comprised an image approved by the Government (Figure 1), i.e. covering 85% of the main display areas, and comprising 60% pictorial area and 25% text on the pack. Compliance with Section 8 was carried out by measuring the entire length and width of the warning (text and pictorial together) for cuboid packs and just the length for cylindrical packs. The colour of the ‘warning’ and the warning message and their background were noted. A clearly conspicuous and legible warning is one that is at least 4 cm long and 3.5 cm wide. Clarity was based on the ability to distinguish the warning from the background. In addition, the text warning must be on a contrasting background (i.e. the term WARNING must be in white font on red background and ‘Tobacco causes cancer’ must be in white font on black background). Compliance with Section 9 was based on the language used for the product name and the text warning. The pack was considered compliant if both were in the same language. Packs were deemed to be noncompliant if the text or pictorial message was split, i.e. only part of the text or pictorial message was seen on the tobacco pack (Figure 2).

Awareness of health warnings on tobacco use was evaluated through a survey of a sample of students in a degree college in Karkala block of Udupi District. Permission was obtained from the principal of the college and a feasible date and time for the survey was decided. The first-year and second-year Arts and Commerce Bachelor degree students aged 18 years and older who were present on the day of the survey were deemed eligible to participate, and all students were briefed regarding the study and given a written participant information sheet. Written informed consent was then collected from those students who agreed to participate in the study. All consenting students were then asked to complete a semi-structured questionnaire that collected information on sociodemographic status, tobacco use among participants, their opinion regarding existing health warnings on tobacco products and impact of health warnings at the time of survey. Tobacco use was defined as having used one or more tobacco products at least once in their lifetime. The questions that were used to assess reactions to the health warning labels were: 1) ‘What do you feel on seeing the pictorial warning?’, and 2) ‘What do you feel on reading the text warning?’. The reactions to health warning labels were assessed with options such as ‘feeling scared’, ‘do not feel like starting’, ‘feel like reducing’, ‘feel like quitting’, and ‘feel nothing’.

Ethics approval for the study was granted by the Institutional ethics committee of Kasturba Hospital, Manipal (IEC: 842/2017).

### Data analysis

Data were entered and analysed using SPSS version 15.0. Basic descriptive procedures were run for the type of tobacco products used, with frequencies and percentages calculated. Means and standard deviations were calculated for age. Chi-squared tests were used to compare measures of compliance, awareness of health warnings on tobacco products and impact of health warnings on preventing tobacco uptake, between males and females.

## RESULTS

### Compliance of the health warnings on tobacco products with COTPA

We obtained 26 tobacco packs (17 from an urban and 9 from a rural locality), comprising six cigarette packs, two beedi packs and 18 smokeless tobacco packs. Two packs (8%) were compliant with Section 7 of COTPA, 15 (58%) were compliant with Section 8 and 18 (69%) with Section 9. The pictorial health warning was split in four packs (15%) and unclear in eleven (42%) packs. One pack (4%) did not have an approved pictorial health warning. The text warning was clear on all packs but split in one pack (4%). Two cigarette packs were compliant with all sections of COTPA.

### Awareness and perceptions of health warnings among college students

On the day of the survey, 81 students were available in the classroom and all agreed to participate in the study. The mean age of the participants was 18.3 years with 35 (43%) males and 46 (57%) females. There were four male and one female tobacco users. Among the study subjects, 46% had observed health warnings on tobacco packs and had seen them on the front or back of the pack. About 32% of the study subjects felt that both pictorial and text warnings were needed to become discouraged from smoking. The response regarding the perception on seeing the four health warnings (Figure 1) was uniform and a majority, up to 70%, felt scared and would not start tobacco consumption but only few of them (4%) felt that they would reduce or quit using tobacco. With respect to the impact of warnings, 21 (60.0%) males and 24 (52.2%) females thought they may be effective in preventing tobacco uptake.

## DISCUSSION

Although based on very small sample sizes, our study provides evidence of substantial non-compliance of tobacco products sold in this district of India with respect to Sections 7, 8 and 9. Of 26 tobacco packs collected, only two were compliant with all three relevant sections of COTPA. Our student survey suggests that the warnings had been seen by less than half of the participants, though a majority thought that the warnings, when present, were effective in discouraging initiation of tobacco use. Only a very small proportion believed that the warnings would promote cessation or reduction of tobacco use in current users.

Our results on health warning compliance complement two other studies in India. The first, by Smith et al.^7^, in which 29 (53%) cigarette packs, no bidis and one smokeless tobacco product were fully compliant with the health warning legislation, and a study by Gupta et al.^8^, where 67% of cigarette brands, 16% of bidi brands, and 46% of smokeless brands were fully compliant with COTPA. The reason for the lower compliance in Karnataka is not clear. There is, however, clearly an urgent need to improve health warning compliance, and particularly for the bidi and smokeless tobacco products that are so widely used in India^2^.

Study limitations include the reliance on convenience store sampling and the small sample sizes, for both the pack collection and survey. It is also possible that there was response bias among survey participants, who were recruited from only a single college. In addition, the survey questions on warnings have not been validated.

Previous research into awareness of health warnings on tobacco products in India has been carried out among the general public^10^, tobacco users^11^ and hospital patients^12^, but these studies all pre-date the introduction of 85% health warnings in 2016. Our study found lower levels of awareness of health warnings than previous studies in urban areas^10,12^, and a much lower proportion believing that the warnings would be effective in stopping tobacco use than in a previous report^10^. However, this latter comparison may be due to the much lower proportion of current users in our sample.

## CONCLUSIONS

Health warnings are one of the most cost-effective methods to communicate the harmful effects of tobacco use in both users and non-users. Our study indicates that the compliance in the district sampled is very low and demands stringent measures to be taken to make the health warnings on tobacco packs better compliant to the National Law. Among the study population, nearly half were aware of the health warnings and felt that they were effective in preventing the non-user from initiating tobacco use.

## References

[1] World Health Organization (2017). WHO report on the global tobacco epidemic: monitoring tobacco use and prevention policies.

[2] World Health Organization, Ministry of Health & Family Welfare Government of India Global adult tobacco survey: GATS-2 India 2016-17.

[3] John RM, Rout SK, Kumar BR, Arora M (2014). Economic burden of tobacco related diseases in India.

[4] Bhojani U Tobacco use in Karnataka: a public health success story in the making.

[5] Ministry of Law and Justice (2003). The cigarettes and other tobacco products (Prohibition of advertisement and regulation of trade and commerce, production, supply and distribution) act, 2003. The Gazette of India.

[6] Ministry of Health & Family Welfare Government of India (2014). Cigarettes and other Tobacco Products (Packaging and Labelling) (Amendment) Rules. The Gazette of India.

[7] Smith K, Welding K, Saraf S, Washington C, Iacobelli M, Cohen J (2018). Tobacco packaging in India: assessing compliance with Health Warning Label (HWL) laws and marketing appeals for cigarettes, bidis and smokeless products. Tob Induc Dis.

[8] Gupta S, Mathew B (2018). Observational study to check the compliance of implementation of 85% graphic health warnings on tobacco products in India from April 1, 2016. Tob Induc Dis.

[9] Tobacco Pack Surveillance System (TPackSS) (2013). India Health Warning Label Compliance Codebook 2013.

[10] Kumar A, Puranik MP (2017). Pictorial health warnings on tobacco packs - a knowledge, attitude and practice survey among Indian engineering students. Int J Health Sci Res.

[11] Dahiya P, Kamal R, Gupta R, Bhatt S, Didhra G, Bansal V (2017). Assessment of awareness about pictorial warnings on tobacco products in tobacco users in Paonta Sahib, Himachal Pradesh, India. Arch Med Health Sci.

[12] Vanishree N, Narayan RR, Naveen N, Bullapa D, Vignesh D, Raveendran NM (2017). Impact of pictorial warning labels on tobacco products among patients attending outpatient department of a dental college in Bangalore city : A cross sectional study. Indian J Cancer.

